# Brote de *Salmonella enterica* subsp. *enterica* serovar Give asociado con enfermedad transmitida por alimentos en Vichada, Colombia, 2015

**DOI:** 10.7705/biomedica.5206

**Published:** 2020-08-31

**Authors:** Nancy Yaneth Flórez, Stefany Alejandra Arévalo, Edna Catering Rodríguez, Jaime Guerrero, Kelly Paola Valverde, Paula Lucía Díaz, Lucy Angeline Montaño, Doris Mabel Gartner, Carolina Duarte, Jaime Enrique Moreno

**Affiliations:** 1 Grupo de Microbiología, Dirección de Investigación en Salud Pública, Instituto Nacional de Salud, Bogotá, D.C., Colombia; 2 Grupo de Microbiología, Dirección de Redes en Salud Pública, Instituto Nacional de Salud, Bogotá, D.C., Colombia; 3 Laboratorio de Microbiología de Alimentos y Bebidas, Instituto Nacional de Vigilancia de Medicamentos y Alimentos, Bogotá, D.C., Colombia; 4 Grupo Evaluación de Riesgos en Inocuidad de Alimentos, Dirección de Vigilancia y Análisis del Riesgo en Salud Pública, Instituto Nacional de Salud, Bogotá, D.C., Colombia; 5 Laboratorio Departamental de Salud Pública, Secretaría de Salud de Vichada, Puerto Carreño, Colombia

**Keywords:** *Salmonella*, brotes de enfermedades, enfermedades transmitidas por los alimentos, vigilancia epidemiológica, Colombia, *Salmonella*, disease outbreak, foodborne illness, epidemiological monitoring, Colombia

## Abstract

**Introducción:**

*Salmonella enterica* subsp. *enterica* serovar Give se encuentra en mamíferos rumiantes, cerdos, aves y ambientes acuáticos, pero rara vez en humanos. En Colombia este serotipo ocupó el decimoprimer lugar en frecuencia en la vigilancia por laboratorio de la enfermedad diarreica aguda entre el 2000 y el 2013.

**Objetivo:**

Caracterizar el fenotipo y el genotipo de *S.* Give en aislamientos relacionados con un brote de enfermedad transmitida por alimentos en el departamento de Vichada en la quinta semana epidemiológica del 2015.

**Materiales y métodos:**

Se buscó *Salmonella* spp. en 37 muestras de materia fecal con el método de estudio del Instituto Nacional de Salud. La muestra de sardinas enlatadas fue procesada según la norma ISO6579:2002 Cor.1:2004. Se determinó el serotipo en los aislamientos confirmados mediante serología o PCR en tiempo real, y se hicieron pruebas de sensibilidad a antimicrobianos y electroforesis en gel de campo pulsado con las enzimas *Xba*l y *Bln*I.

**Resultados:**

Todos los aislamientos de origen humano (11) y el aislamiento del alimento (1), se identificaron como *S.* Give y este último presentó resistencia a la tetraciclina. El análisis por PFGE-*Xba*I agrupó bajo el patrón COIN15JEXX01.0005 diez aislamientos de origen humano y a los restantes bajo el COIN15JEXX01.0006, con un 96,3% de similitud. Los resultados de todos los aislamientos se confirmaron con la enzima *Bln*I; cuatro de ellos (tres humanos y el del alimento) se agruparon bajo el patrón COIN15JEXA26.002, con un porcentaje de similitud del 95,65%.

**Conclusión:**

El estudio confirmó que las sardinas enlatadas se relacionaron con la transmisión de *S.* Give en el brote, que es el tercero ocasionado por este serotipo en Colombia.

## Abstract (en)

**Introduction:**

*Salmonella enterica* subsp. *enterica* serovar Give is found in ruminants, pigs, poultry, and aquatic environments, but rarely in humans. In Colombia, this serotype was ranked 11^th^. in the laboratory surveillance of acute diarrheal disease between 2000 and 2013.

**Objective:**

To characterize phenotypic and genotypic isolates of *Salmonella* related to an outbreak of foodborne Illness in the department Vichada in the fifth epidemiological week of 2015.

**Materials and methods:**

Following the *Instituto Nacional de Salud* method, we tested 37 fecal samples for *Salmonella* spp. while the sample of canned sardines was processed according to the ISO 6579:2002 Cor.1:2004 standard. The isolates were confirmed by serology and/or real-time PCR, antimicrobial susceptibility tests, and pulsed-field gel electrophoresis with the *Xba*I and *Bln*I enzymes.

**Results:**

All human isolates (11) and that from food (1) were identified as *S.* Give. The food isolate exhibited tetracycline resistance. PFGE analysis with *Xba*I grouped ten isolates from samples of human origin in pattern COIN15JEXX01.0005 and the remaining isolates in COIN15JEXX01.0006 with 96.3% similarity. All isolates were confirmed with the *Bln*I enzyme, and four (three human isolates and the one from food) were matched to the pattern COIN15JEXA26.002 with 95.65% similarity.

**Conclusion:**

Our study confirmed that canned sardines were related to the transmission of *S.* Give in the outbreak, which is the third one caused by this serotype in Colombia.

Las enfermedades transmitidas por los alimentos constituyen un problema de salud pública creciente a nivel mundial y su incidencia es difícil de estimar. En el 2015, la Organización Mundial de la Salud (OMS) señaló que se enferman anualmente unos 600 millones de personas en el mundo (uno por cada diez habitantes) al ingerir alimentos contaminados con virus, bacterias, parásitos o agentes químicos y, en el 2010, se reportaron cerca de 420.000 muertes. Los niños menores de cinco años representaron el 40% (125.000 fallecidos) de la mortalidad atribuible a dichas enfermedades y se estima que 230.000 muertes fueron ocasionadas por *Salmonella enterica* no tifoidea (1).

En el 2015, en Colombia, se notificaron al Sivigila 10.243 casos de enfermedades transmitidas por alimentos en 858 brotes; el grupo de edad más afectado fue el de 10 a 14 años, con una tasa de morbilidad a nivel nacional de 21,01 casos por 100.000 habitantes. Del total de brotes con agente etiológico identificado, 162 provenían de muestras de alimentos o de agua y de superficies, y en 119 muestras biológicas se identificaron como agentes causales bacterias (*Staphyloccus aureus*, *Escherichia coli*, *Salmonella* spp., *Bacillus cereus*, *Listeria monocytogenes*, *Campylobacter* spp., *Shigella* spp. y *Aeromonas hydrophila*), virus, parásitos y compuestos organofosforados (2).

Entre los serotipos de *Salmonella* spp. causantes de enfermedades transmitidas por alimentos, se encuentra *Salmonella enterica* subsp. *enterica* serovar Give, el cual se relaciona con mamíferos rumiantes y animales para el consumo, pero rara vez con huéspedes humanos (3). En 1996, la Red Nacional de Vigilancia Epidemiológica de España reportó la presencia de *S.* Give en 3 de 881 aislamientos no humanos: uno de alimento (carne) y dos del ambiente (agua de río y de mar) (4). En Francia, en el año 2008, se presentó un brote de *S.* Give en lactantes asociado con el consumo de leche en polvo (5). En el 2012, Borriello, *et al.,* reportaron una prevalencia del 25% de *Salmonella* spp. en terneros de búfalo acuático (*Bubalus bubalis*) con gastroenteritis y en las muestras se destacó *S.* Give (11%) (6). En el 2015, en los Estados Unidos, Maurer, *et al*. estudiaron muestras de agua y mamíferos pequeños en dos áreas geográficas (planicie costera y piedemonte), y encontraron 37 serotipos, entre ellos *S.* Give, en las dos cuencas (7).

En el marco de la vigilancia por laboratorio de la enfermedad diarreica aguda y la transmitida por alimentos en Colombia, el Grupo de Microbiología del Instituto Nacional de Salud caracteriza los aislamientos de *Salmonella* spp. remitidos por los laboratorios de salud pública; al analizar la distribución de los aislamientos clínicos por serotipos entre el 2000 y el 2013, *S.* Give ocupó el decimoprimer lugar de frecuencia, con 96 aislamientos (1,3%) de un total de 7.219; en cuanto a la distribución de los aislamientos provenientes de muestras de alimentos enviadas al Instituto Nacional de Salud, *S.* Give fue el decimoctavo, con dos aislamientos provenientes de carne de res y queso correspondientes al 1,0% de un total de 205, según datos sin publicar del Grupo de Microbiología (8).

En este contexto, se presenta el estudio de un brote de enfermedad transmitida por alimentos en una población humana, causado por *S.* Give en el departamento de Vichada en la quinta semana epidemiológica del 2015.

## Materiales y métodos

### Descripción del brote

El día 26 de enero de 2015 se presentó un brote de enfermedad transmitida por alimentos entre los trabajadores de una finca agrícola ubicada en la zona rural del municipio de La Primavera, departamento de Vichada, Colombia. Ochenta personas consumieron diversos alimentos, entre los que se incluían sardinas enlatadas, pollo y agua de acequia; 45 de ellos desarrollaron síntomas y se remitieron 37 muestras de materia fecal al Grupo de Microbiología del Instituto Nacional de Salud para su estudio. Los casos se informaron al Sistema Nacional de Vigilancia en Salud Pública (Sivigila) en las fichas de notificación para enfermedades transmitidas por alimentos (código INS): 355 como notificación individual y 350 como notificación colectiva.

### Investigación de campo

La información sobre el evento la recogió la Secretaría de Salud de Vichada en el “Anexo 2 ETA, encuesta a consumidores”, siguiendo los criterios establecidos por el Instituto Nacional de Salud (http://www.ins.gov.co/Direcciones/Vigilancia/Paginas/Lineamientos-y-documentos.aspx). En esta encuesta se compilan datos sociodemográficos como edad y sexo, fecha de notificación del caso, signos y síntomas, lugar de consumo y alimentos consumidos, entre otros.

Durante la visita de inspección, vigilancia y control del evento, se aplicaron medidas sanitarias y se relacionaron los siguientes alimentos asociados con el brote: latas de sardinas, pollo y agua de acequia, aunque solo se recolectaron muestras de las latas de sardinas, las cuales se remitieron al Laboratorio Departamental de Salud Pública y al Instituto Nacional de Vigilancia de Medicamentos y Alimentos (Invima) para los análisis microbiológicos.

El Grupo de Microbiología del Instituto Nacional de Salud recibió 37 muestras de materia fecal en medio de transporte Cary-Blair (BD BBL, USA) provenientes del Laboratorio de Salud Pública de Vichada, para la identificación del agente etiológico y su posterior caracterización fenotípica y genotípica.

### Caracterización fenotípica y genotípica de las muestras

*Aislamiento bacteriano.* Se analizaron 37 muestras de materia fecal preservadas en medio de transporte Cary-Blair con el método de estudio del Instituto Nacional de Salud (MEN-R01.5330-002). Se hizo el preenriquecimiento de las muestras en caldo selenito y se incubaron a 35 ± 2 °C durante ocho horas; posteriormente, se sembraron por agotamiento en los medios selectivos de xilosa, lisina, desoxicolato (XLD) y Hecktoen (HE), y se incubaron a 35 ± 2 °C durante 24 horas. La identificación se hizo empleando el equipo semiautomatizado MicroScan AutoScan-4™ con el panel NUC60 (Siemens).

En cuanto a las muestras de alimentos, según las normas sanitarias vigentes, los alimentos enlatados deben someterse a la prueba de esterilidad comercial, pero, dado que la muestra estaba relacionada con una enfermedad transmitida por alimentos, en este caso se hicieron análisis para *Salmonella* spp. con la metodología descrita en la Norma ISO 6579:2002 cor 1:2004 (9) acreditada ante el Organismo Nacional de Acreditación de Colombia (ONAC), con el fin de seleccionar tres colonias del aislamiento primario.

Una porción de 25 g de la muestra se transfirió asépticamente a una bolsa con 225 ml de agua de peptona con solución tampón y se incubó a 37 ± 1 °C durante 18 a 24 horas para, luego, hacer el enriquecimiento selectivo en caldo Rapapport Vassiliadis incubado a 41,5 ± 1 °C durante 24 ± 3 horas y, en Mueller Kauffmann, con tetrationato a 37 ± 1 °C durante 24 ± 3 horas. Para el aislamiento bacteriano, se emplearon los agares selectivos XLD y HE a 35 ± 2 °C durante 24 horas. Para la identificación de las bacterias, se usó el sistema de pruebas bioquímicas API 20E™.

*Determinación del serotipo.* El serotipo de los aislamientos clínicos de *Salmonella* spp. se determinó mediante PCR en tiempo real (*Multiplex real-time PCR,* MRT-PCR), siguiendo el protocolo estandarizado por Muñoz, *et al.* en el 2010 (10). En los aislamientos confirmados como grupo E (factor O: 3,10), se determinaron los antígenos flagelares “H” con el esquema de Kauffmann-White-Le Minor (11), evidenciándose floculación en medio líquido (12,13), en tanto que, para el aislamiento del alimento, solo se empleó el esquema de Kauffmann-White-Le Minor.

*Sensibilidad antimicrobiana.* En los aislamientos recuperados de muestras clínicas, se analizó la sensibilidad antimicrobiana a 25 antibióticos con el equipo semiautomatizado MicroScan AutoScan-4™ (Siemens) y el panel NUC60 (Siemens).

La sensibilidad antimicrobiana de los aislamientos recuperados de muestras del alimento, se determinó con el panel NMIC/ID-132 (Becton Dickinson and Company), en el que se evaluaron 20 antibióticos utilizando el equipo Phoenix 100™ (Becton Dickinson and Company).

*Caracterización molecular de Salmonella* spp. Los aislamientos identificados como *S.* Give se analizaron por electroforesis en gel de campo pulsado (PFGE) con las enzimas de restricción *Xba*l (Promega, USA) y *Bln*I (Roche) para determinar su perfil genómico mediante el patrón de bandeo y siguiendo el protocolo de la Red PulseNet (CDC, Atlanta). Como marcador de peso molecular, se empleó *Salmonella* Braenderup H9812, en tanto que los geles se analizaron con el programa Gel Compare II, versión 4.0, empleando el coeficiente de Dice, algoritmo de emparejamiento de bandas basado en promedios aritméticos-UPGMA, con lo que se obtuvieron los dendrogramas respectivos (14). El patrón de bandas se comparó con la Base de Datos Regional para América Latina y Caribe en el Instituto Nacional de Enfermedades Infecciosas A.N.L.I.S. “Dr. Carlos G. Malbrán”, Centro Regional de Referencia del *WHO Global Salm Surv*.

## Resultados

### Descripción del brote

**Figura 1:**
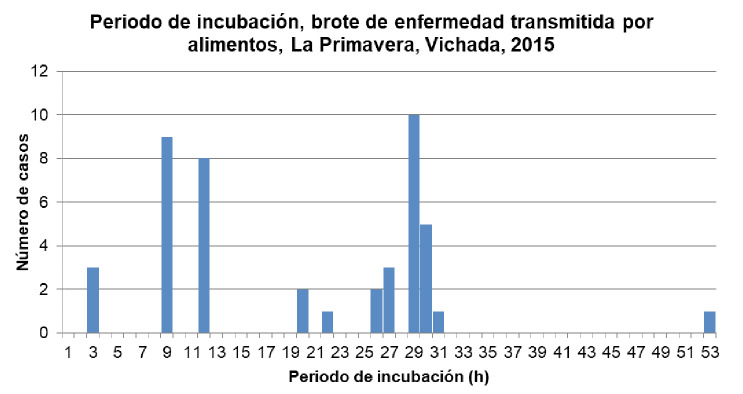
Periodo de incubación en un brote de enfermedad transmitida por alimentos, La Primavera, Vichada, 2015 (h): horas

El primer caso fue reportado el 26 de enero de 2015 y, el último, dos días después. La información obtenida mediante la encuesta a consumidores permitió detectar en la curva epidémica 3 horas como el periodo de incubación más corto y 53 horas como el más largo, y a las 29 horas se presentó el periodo con el mayor número de casos (n=10) (figura 1). El 95,5% de los afectados presentó diarrea, el 84,4% vómito, y el 80% náuseas y deshidratación, entre otros síntomas (cuadro 1). El análisis de distribución por grupos de edad demostró que el mayor número de afectados entre las 45 personas relacionadas con el brote, se encontraba entre los 20 y los 24 años (20%), y el 93,3% de la población era de sexo masculino (cuadro 2).

**Cuadro 1:** Distribución de signos y síntomas en un brote de enfermedad transmitida por alimentos, La Primavera, Vichada, 2015

**Síntomas**	**n**	**(%)**
Diarrea	43	95,6
Vómito	38	84,4
Náuseas	36	80,0
Deshidratación	36	80,0
Fiebre	30	66,7
Cefalea	21	46,7
Artralgias	14	31,1
Calambres abdominales	12	26,7
Mialgias	11	24,4
Mareos	11	24,4

**Cuadro 2:** Porcentaje de enfermos por grupo de edad en un brote de enfermedad transmitida por alimentos, La Primavera, Vichada, 2015

**Grupos de edad (años)**	**Total**	**Hombres**		**Mujeres**	
15 a 19	3	2		1	
20 a 24	9	9		0	
25 a 29	7	7		0	
30 a 34	3	2		1	
35 a 39	3	3		0	
40 a 44	7	6		1	
45 a 49	7	7		0	
50 a 54	1	1		0	
55 a 59	3	3		0	
>60 años	2	2		0	
Total	45	42		3	

### Investigación de campo

De los 45 trabajadores afectados por el brote, 25 fueron atendidos en el hospital local del municipio de La Primavera, el 79% estuvo en observación y no requirió hospitalización y el otro 21% no necesitó ningún procedimiento médico. Los 20 pacientes restantes fueron atendidos en el hospital local del municipio de Santa Rosalía; de ellos, el 65% fue hospitalizado y los demás fueron atendidos por consulta externa, se rehidrataron y se mantuvieron en observación.

El reporte de la situación sanitaria en el lugar determinó varios incumplimientos a lo establecido en las normas vigentes (Decreto 3075 de 1997 y Resolución 2674 de 2013) en relación con las condiciones básicas de higiene en la fabricación de alimentos, edificación de instalaciones, equipos y utensilios, personal manipulador de alimentos y saneamiento. Los alimentos provenían de la ciudad de Villavicencio (Meta) y fueron transportados sin las condiciones adecuadas de conservación. Por ello, los organismos de vigilancia y control tomaron medidas de seguridad en el establecimiento, como la suspensión parcial o total de la fabricación de alimentos y la destrucción total del alimento implicado.

Asimismo, se elaboró un plan de mejora de la infraestructura con los responsables del establecimiento y se ofreció capacitación en buenas prácticas de manufactura al personal manipulador residente en los municipios de Puerto Carreño y La Primavera. Los compromisos adquiridos se verificaron en las visitas de seguimiento a cargo de las entidades competentes.

### Caracterización fenotípica de los aislamientos

El 29,7% (11/37) de las muestras de origen humano resultó positivo para *Salmonella* spp. La PCR en tiempo real arrojó resultados positivos para el antígeno somático E (factor O: 3,10) y, con el esquema de Kauffmann-White-Le Minor, se determinaron las fases flagelares l,v: 1,7 correspondientes al serotipo Give (fórmula antigénica 3,10: I,v: 1,7). Los aislamientos fueron sensibles a los antimicrobianos evaluados (MicroScan™), entre ellos, tetraciclina, trimetroprim- sulfametoxasol, ampicilina, cefotaxima y ceftazidima, todos considerados de gran importancia en salud pública.

Las tres colonias analizadas del aislamiento primario obtenido de una lata de sardinas, se identificaron como *Salmonella* spp. y, por serología, como *S.* Give (fórmula antigénica 3,10: I,v: 1,7), con resistencia a tetraciclina. No se detectaron otros agentes patógenos en el alimento estudiado.

### Caracterización molecular

**Figura 2:**
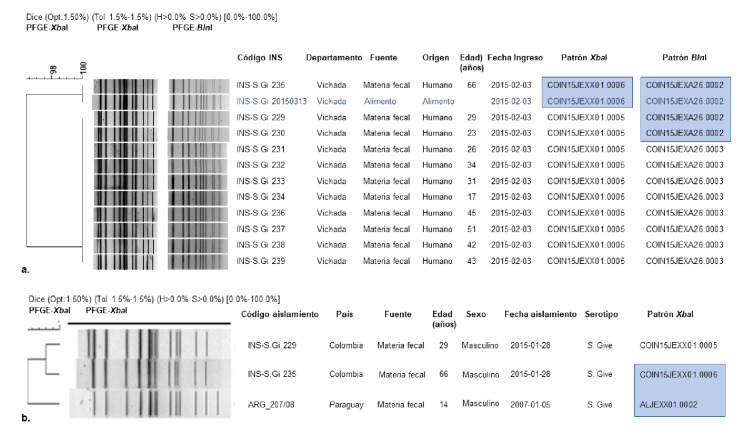
**a.** Dendrograma PFGE *Xba*I-*Bln*I de la relación genética de los aislamientos del brote de *S.* Give, municipio La Primavera, departamento de Vichada, Colombia, 2015. **b.** Dendrograma PFGE *Xba*I de comparación con la Base de Datos Regional de PulseNet en América Latina y el Caribe para establecer la relación genética de los aislamientos del brote de *S.* Give en el municipio La Primavera, departamento de Vichada, Colombia, 2015 Descripción del código patrón de PFGE: COIN: Instituto Nacional de Salud; 15: año 2015; JEX: *Salmonella* Give; XO1: enzima *Xba*I; A26: enzima *Bln*I; 0005, 000

El análisis comparativo mediante PFGE de 11 aislamientos humanos y uno de alimento con la enzima *Xba*I mostró el patrón COIN15JEXX01.0005 con 13 bandas, el cual agrupó 10 de los 11 aislamientos de humanos (90,9%). El aislamiento de origen humano restante presentó el patrón COIN15JEXX01.0006 de 12 bandas y se obtuvo una similitud genética del 96% entre los dos grupos mencionados. El aislamiento de las sardinas enlatadas correspondió al patrón COIN15JEXX01.0006. El análisis por PFGE con la enzima secundaria *Bln*I evidenció dos patrones, el COIN15JEXA26.0002, con 11 bandas, que agrupó cuatro aislamientos (tres provenientes de humanos y el del alimento), y el COIN15JEXA26.0003, con 12 bandas, que agrupó los ocho aislamientos humanos restantes, con un 95,65% de similitud genética (figura 2a.). Estos resultados indicaron que los 12 aislamientos estudiados estaban estrechamente relacionados y hacían parte del mismo brote. La relación encontrada entre el genotipo aislado de las sardinas enlatadas y los aislamientos de origen humano, permitió establecer este alimento como la fuente de infección con *S.* Give.

La comparación del patrón único COIN15JEXX01.0006 correspondiente a un aislamiento de origen humano de *S*. Give con la base de datos regional mostró un 90,61% de similitud con un aislamiento del mismo origen proveniente de Paraguay (ARG_207/08) y remitido en el 2008 (patrón único de bandas ALJEXX01.0002) (figura 2b).

## Discusión

*Salmonella enterica* subsp. *enterica* serovar Give se ha relacionado principalmente con mamíferos rumiantes, cerdos, aves silvestres y ambientes acuáticos, pero, rara vez, con huéspedes humanos (3,4,6,15). Entre los brotes conocidos en humanos, se encuentran el ocurrido en varios estados de Alemania en el 2004, asociado con el consumo de cerdo crudo picado (16), y el de Francia en el 2008 en lactantes, asociado con el consumo de leche en polvo (5).

Según la base de datos de la vigilancia por laboratorio que adelanta el Grupo de Microbiología del Instituto Nacional de Salud desde 1997, este es el tercer brote causado por *S.* Give en una población colombiana. Los reportados previamente en el país se presentaron en los departamentos de Huila en el 2008 y Quindío en el 2012 y, en ninguno de los casos, se recolectó el alimento implicado. En el primero se vieron afectadas 23 personas y el análisis por PFGE de 11 aislamientos exhibió los patrones únicos COIN.JEX.X01.0001 para la enzima *Xba*I y COIN.JEX.A26.0001 para la enzima *BlnI* (8). El segundo brote afectó a tres personas y se identificó el patrón único para la enzima *Xba*I COIN12JEXX01.0006 (Grupo de Microbiología, Instituto Nacional de Salud, datos sin publicar).

Dado que el patrón de PFGE-*Xba*I COIN15JEXX01.0006 relacionó un aislamiento humano y el recuperado de las sardinas enlatadas con una sola banda de diferencia, los aislamientos se clasificaron como estrechamente relacionados con la cepa del brote (aislamiento del alimento) según los criterios aceptados para su confirmación (17), lo que fue corroborado con el análisis comparativo de PFGE-*Bln*I (patrón COIN.JEX.A26.0001). El análisis de PFGE por serotipo con las dos enzimas de restricción *Xba*I y *Bln*I, que reconocen y cortan el ADN en sitios específicos (secuencias TCTAGA y CCTAGG, respectivamente) (18), permite establecer con mayor exactitud las relaciones genéticas entre los aislamientos de un brote y el seguimiento epidemiológico molecular (19,20).

Los resultados del presente estudio evidenciaron una relación estrecha entre la población afectada y el consumo de las sardinas enlatadas, relación ya sugerida por la investigación de campo, por lo que es posible inferir que los aislamientos estudiados probablemente son parte de este brote; además, el patrón de restricción de PFGE del alimento se designó como patrón del brote por nexo epidemiológico. Probablemente, la diferencia en los patrones únicos obtenidos con las enzimas de restricción se deba a una mutación esporádica en la cepa del brote (17,21).

El patrón PFGE-*Xba*I COIN15JEXX01.0006 identificado en las sardinas y en uno de los aislamientos clínicos, coincidió con los aislamientos provenientes del brote ocurrido en el 2012 en el Quindío, lo que sugiere la circulación de este patrón en el tiempo y en otras regiones del país, que puede atribuirse a su naturaleza ambiental y zoonótica, lo cual le permite transmitirse de animales a humanos (7).

En el marco de la vigilancia de alimentos realizada por el laboratorio del Invima desde el 2011, *S.* Give representa el 1,4% del total de serotipos aislados y se encuentra asociado con muestras de cárnicos (datos sin publicar). La elaboración y el empaque de los productos enlatados están sujetos a controles estrictos, sin embargo, su conservación es fundamental para mantener su calidad.

Generalmente, las sardinas enlatadas se han relacionado con contaminantes no biológicos (22-24), por lo que este es el primer reporte de contaminación de un producto enlatado por *S.* Give en el país, lo que se relacionó directamente con su estado de conservación, pues se evidenciaron filtraciones y abolladuras en el empaque y, dado que el lugar de producción no estaba protegido frente a la entrada de contaminantes físicos (aire, agua, polvo) ni biológicos (silvestres y domésticos), es posible inferir que la contaminación de la lata de sardinas por *S*. Give provenía del contacto directo con alguna especie animal infectada (no identificada), o que actuó como vehículo de contaminación ambiental, lo cual concuerda con la asociación ya establecida de *S.* Give con animales y ambientes acuáticos (3,4,6,7,25-29).

El análisis de los resultados epidemiológicos, microbiológicos y moleculares, permite señalar que la fuente más probable de infección con *S.* Give en los trabajadores afectados por el brote fueron las sardinas enlatadas; sin embargo, no fue posible determinar la forma en que el alimento se contaminó con este serovar. Con excepción del aislamiento proveniente del alimento resistente a tetraciclina, todos los de origen humano fueron sensibles a los antimicrobianos del panel NUC60, hecho atribuible a los bajos niveles de exposición que no inducen una reacción adaptativa de intercambio o adquisición de genes de resistencia (30). *S.* Give fue el principal serovar encontrado en un estudio realizado en Costa Rica con altas tasas de resistencia a tetraciclina en un producto utilizado para alimentación animal a base de harina de carne y huesos (31). En otros estudios se ha documentado, además, la resistencia a cefalosporinas y monobactámicos (32).

La Base de Datos Regional de Patrones de PFGE de la red PulseNet en Latinoamérica y el Caribe (PNLAC), reúne aproximadamente 8.600 casos y brotes en los países miembros. Este registro ha permitido fortalecer la vigilancia nacional y regional con fines de prevención, control e investigación de brotes (33); en ese contexto, los dos patrones de PFGE-*Xba*I obtenidos fueron compartidos con la red. En el análisis comparativo de los patrones del brote se encontró similitud con un aislamiento de Paraguay (90,6%) del 2007, lo que permite suponer una posible relación genética entre los aislamientos.

Este es el primer reporte de un brote por contaminación con *S.* Give en un producto enlatado en el país. Su estudio contribuye al conocimiento de este serovar como un agente causante de brotes de enfermedades transmitidas por alimentos, así como de las medidas aplicadas para controlar y prevenir su propagación en la población afectada.
